# Caenorhabditis Elegans Exhibits Morphine Addiction-like Behavior via the Opioid-like Receptor NPR-17

**DOI:** 10.3389/fphar.2021.802701

**Published:** 2022-01-03

**Authors:** Soichiro Ide, Hirofumi Kunitomo, Yuichi Iino, Kazutaka Ikeda

**Affiliations:** ^1^ Addictive Substance Project, Tokyo Metropolitan Institute of Medical Science, Tokyo, Japan; ^2^ Department of Biological Sciences, Faculty of Science, Graduate School of Science, The University of Tokyo, Tokyo, Japan

**Keywords:** *C. elegans*, morphine, preference, addiction, NPR-17

## Abstract

Addiction has become a profound societal problem worldwide, and few effective treatments are available. The nematode *Caenorhabditis elegans* (*C. elegans*) is an excellent invertebrate model to study neurobiological disease states. *C. elegans* reportedly developed a preference for cues that had previously been paired with addictive drugs, similar to place conditioning findings in rodents. Moreover, several recent studies discovered and reported the existence of an opioid-like system in *C. elegans*. Still unclear, however, is whether *C. elegans* exhibits addictive-like behaviors for opioids, such as morphine. In the present study, we found that *C. elegans* exhibited dose-dependent preference for morphine using the conditioned chemosensory-cue preference (CCP) test. This preference was blocked by co-treatment with the opioid receptor antagonist naloxone. *C. elegans* also exhibited aversion to naloxone-precipitated withdrawal from chronic morphine exposure. The expression of morphine-induced CCP and morphine withdrawal were abolished in worms that lacked the opioid-like receptor NPR-17. Dopamine-deficient mutant (*cat-2 (e1112)*) worms also did not exhibit morphine-induced CCP. These results indicate that the addictive function of the opioid system exists in *C. elegans*, which may serve as a useful model of opioid addiction.

## Introduction

Addiction, including both substance use disorder (e.g., stimulants, opioids, and alcohol) and behavioral addiction (e.g., gaming and gambling), affects the mind, body, and social activities of the sufferer and has become a profound societal problem worldwide. In the United States alone, nearly 70,000 people died from opioid-involved overdoses in 2020, underscoring the so-called “opioid crisis.” Thus, a better understanding of the neurobiological basis of addiction and the discovery of more effective treatments are needed. Although the development of animal models of drug reward and addiction, especially using rodent models, has provided much of our current understanding about the neuroscience of addiction (Kuhn et al., 2019), this is still not sufficient to solve the problem. The nematode *Caenorhabditis elegans* (*C. elegans*) has evolutionarily conserved neurobiological systems with the established mapping of all neurons and synapses in the entire animal. It is thought to have major advantages as a model organism to study neurobiology and disease states (Hulme and Whitesides, 2011). *C. elegans* was also proposed to be used to model aspects of drug addiction (Engleman et al., 2016). *C. elegans* was reported to develop a conditioned preference for cues (salt or food cue) that had previously been paired with either cocaine or methamphetamine exposure via dopaminergic neurotransmission (Musselman et al., 2012; Katner et al., 2016). Furthermore, *C. elegans* reportedly exhibited preference for not only these psychostimulants but also other addictive substances, including alcohol and nicotine (Lee et al., 2009; Sellings et al., 2013; Engleman et al., 2018). Still unclear, however, is whether *C. elegans* exhibits a preference for opioids, such as morphine. Naloxone (NLX) is well known to cause withdrawal symptoms in rodents, such as aversion in chronic opioid-treated animals, but unclear is whether such withdrawal symptoms can be observed in nematodes. Opioids are the oldest drugs and have diverse beneficial and deleterious effects, ranging from pain relief to addiction. The endogenous opioid system in mammals is composed of μ-, δ-, and κ-opioid receptors and endogenous ligands for these receptors. This system is well known to play important roles in the modulation of pain sensitivity, stress responses, feeding behavior, and addiction, but relatively little is known about whether this system has similar characteristics in lower animals. The treatment of *C. elegans* with morphine and the opioid neuropeptides endomorphin-1 and -2 affected thermal avoidance behavior, and these effects were reversed by the opioid receptor antagonists NLX and CTOP (Nieto-Fernandez et al., 2009). Cheong *et al.* (2015) identified the NLP-24 peptide in *C. elegans*, which was shown to have a conserved YGGXX sequence, similar to mammalian opioid neuropeptides that possess a common N-terminal YGGF signature sequence. These authors also showed that morphine and NLX stimulated and inhibited feeding, respectively, in starved worms but not in worms that lacked the opioid-like receptor NPR-17 (Cheong et al., 2015). Morphine suppressed overall withdrawal from noxious stimuli through a pathway that depends on NPR-17 (Mills et al., 2016). These results indicate that the function of the opioid system also exists in *C. elegans*. However, unknown is whether opioids exert addiction-like effects in *C. elegans*. Thus, we investigated addictive-like effects of morphine in *C. elegans*.

## Materials and Methods

### Strains and Culture

Bristol N2 worms were used as a wildtype strain of *C. elegans*. Worms were cultivated on standard nematode growth medium plates using standard methods (Brenner, 1974). The NPR-17-mutant strain (*tm3210*) was obtained from S. Mitani (Tokyo Women’s Medical University, Tokyo, Japan; National BioResource Project). The dopamine-deficient strain (CB1112: *cat-2(e1112)* mutant (Sulston et al., 1975)) was obtained from the Caenorhabditis Genetics Center. The NA22 *Escherichia coli* (*E. coli*) strain was fed to animals under normal cultivation conditions, and the OP50 *E. coli* strain was fed to animals that were used for the behavioral assays. For age synchronization, four young adult worms that accumulated fertilized eggs in the uterus were chosen for the new OP50/nematode growth medium (NGM) plate. They were then grown for 4 days at 20°C before the assays.

### Conditioned Chemosensory-Cue Preference Test

**FIGURE 1:**
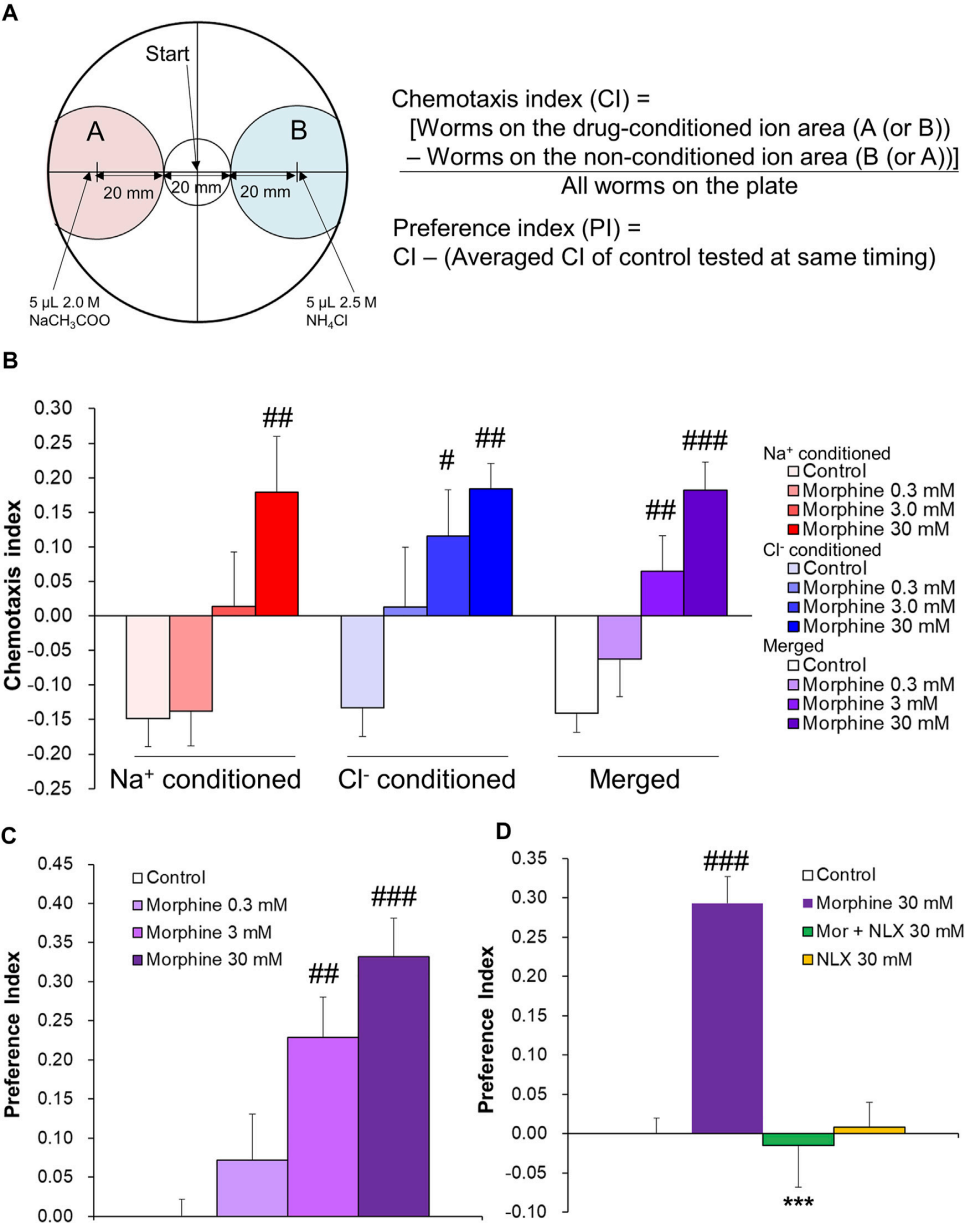
Preference for morphine-conditioned cue in wildtype *C. elegans* in the CCP tests. **(A)** Testing plate and CI and PI formulae. **(B)** Chemotaxis Index after 1 h of morphine conditioning. One-way ANOVA: Na^+^-conditioned, F_3,15_ = 5.52, *p* = 0.009; Cl^−^-conditioned, F_3,15_ = 6.00, *p* = 0.007; merged, F_3,34_ = 10.84, *p* < 0.0001. (control, *n* = 6; 0.3 mM morphine, *n* = 4; 3.0 mM morphine, *n* = 5; 30 mM morphine, n = 4) **(C)** Preference Index after 1 h of morphine conditioning. One-way ANOVA: F_3,34_ = 9.657, *p* < 0.0001. (control, *n* = 12; 0.3 mM morphine, *n* = 8; 3.0 mM morphine, *n* = 10; 30 mM morphine, *n* = 8) **(D)** Preference Index after 1 h of 30 mM morphine and 30 mM naloxone (NLX) conditioning. Two-way ANOVA: effect of morphine, F_1,30_ = 14.84, *p* = 0.0006; effect of NLX, F_1,30_ = 18.26, *p* = 0.0002; interaction, F_1,30_ = 20.22, *p* < 0.0001 (control, *n* = 14; morphine alone, *n* = 8; morphine + NLX, *n* = 6; NLX alone, *n* = 6) ^##^ *p* < 0.01, ^###^ *p* < 0.001, compared with control worms; ***p < 0.001, compared with 30 mM morphine alone-treated worms.

Rewarding effects were evaluated using the conditioned chemosensory-cue preference (CCP) test, which was performed according to the reward assay in Sellings et al. (2013) with modifications. Assay plates were used, consisting of standard 90-mm Petri dishes that contained 10 ml of sodium salt-free BG50 agar (1 mM CaCl_2_, 1 mM MgSO_4_, 25 mM potassium phosphate [pH 6.0], and 20 g/L Bactoagar). Assay plates with a concentration of 75 µM salt (either sodium acetate or ammonium chloride) were used as conditioning plates. For drug pairing, 3 h before conditioning, half of the conditioning plates were spotted with three 5-µl drops of drugs (0.3, 3.0, and 30 mM morphine hydrochloride solution, 30 mM naloxone hydrochloride [NLX] solution, 30 mM morphine +30 mM NLX solution, 50 mM nicotine tartrate solution, or 500 mM methylphenidate [MPH] solution) at 25-mm intervals along the diameter of the plate (the conditioning plates contained a single concentration of an individual drug). Worms were washed three times with assay buffer (1 mM CaCl_2_, 1 mM MgSO_4_, and 25 mM potassium phosphate [pH 6.0]) and then transferred to salt- and drug-free assay plates for 30 min before conditioning for acclimation. The worms were then transferred to the center of the conditioning plates and left for 1 h. Conditioning plates without drugs were used as controls for the assay. After conditioning, the worms were washed three times with assay buffer and transferred to the center of the testing plates. For the testing plates, 3.5 h before use, 5 µl of sodium acetate (2 M) and 5 μl of ammonium chloride (2.5 M) were spotted on opposite ends of the plates, 30 mm from the center, to form two opposing diffusion gradients (Figure 1A). Ninety minutes after transferring the worms to the testing plates, the plates were immediately placed under 4°C conditions for 60 min to suppress worm activity, and then the number of worms in each area was counted. All conditioning experiments for sodium and chloride ions were conducted with the same number of plates (counterbalanced). The Chemotaxis Index (CI) was calculated using the number of worms within 20 mm of either salt according to the following formula ([*Worms in the drug-conditioned ion area*]*—[Worms in the non-conditioned ion area]*)*/(All worms on the plate)* (Figure 1A). *C. elegans* was previously shown to exhibit an aversive response to a cue that had been paired with starvation (Saeki et al., 2001). In the present study, control worms were exposed to sodium or chloride ions during a 1 h conditioning period, during which time they were starved because of a lack of food. Thus, the control worms learned about starvation during conditioning and exhibited aversion to the conditioned ions in the subsequent test session. Drug-treated worms learned about starvation as well, and chemotaxis during the subsequent test session was thought to result from the aversion to starvation and preference for the drug, thereby canceling each other out. When addictive drugs were added, aversion was suppressed, in which aversion to conditioned ions occurred at higher concentrations. Thus, we further calculated the Preference Index (PI) to evaluate the approach response to conditioned cues, with the exception of aversion that was caused by starvation. The PI for each worm was calculated according to the following formula: *CI—(Average CI of control worms tested at same time)*. The raw data of CIs and PIs in each experiment are shown in a dataset.

### Morphine Withdrawal Test

Naloxone-precipitated morphine withdrawal-induced conditioned place aversion was also evaluated using the CCP test. *C. elegans* strains were grown for 4 days at 20°C on 45 µM morphine (final concentration)-containing NGM plates by adding 15 µl of 30 mM morphine to 10 ml NGM with the OP50 *E. coli* feeding strain. The CCP test used conditioning plates that were spotted with three 5-µl drops of 30 mM NLX solution or assay buffer (as a control). The test was performed according to the method described above.

### Statistical Analysis

All experiments were conducted in duplicate (8–28 test plates *per* drug concentration). The results are expressed as mean ± SEM and were analyzed using one-way analysis of variance (ANOVA) followed by Dunnett’s multiple-comparison post hoc test or two-way ANOVA followed by the Sidak multiple-comparison post hoc test. Values of *p* < 0.05 were considered statistically significant.

## Results

### Wildtype Worms Concentration-dependently Prefer a Morphine-Conditioned Cue

A conditioned chemosensory-cue preference (CCP) experiment was first conducted to determine whether *C. elegans* forms associations with chemosensory cues that are paired with morphine. The Chemotaxis Index (CI) and Preference Index (PI) were calculated according to the formula in Figure 1A. The control group exhibited an aversive response to conditioned salts (sodium acetate [Na^+^] or ammonium chloride [Cl^−^]) (Figure 1B). This was consistent with a previous report (Jang, Toyoshima, Tomioka, Kunitomo, and Iino, 2019; Saeki et al., 2001) and suggested that the worms learned and showed aversion to salts that were associated with starvation during the conditioning period. Worms exhibited dose-dependent approach to the morphine-conditioned cues (Na^+^-conditioned, *p* = 0.009; Cl^−^-conditioned, *p* = 0.007; merged, *p* < 0.0001), although Na^+^ conditioning with 3.0 mM morphine tended to have a slightly weaker effect (Figure 1B). We further evaluated the approach response to conditioned cues by calculating the PI, which compensated for aversion that resulted from the association between conditioning ions and starvation that was seen in controls in each test that was conducted at the same time. Morphine treatment dose-dependently increased the PI (*p* < 0.0001; Figure 1C). Next, to confirm that morphine preference resulted from an action on a specific molecule, we examined behavioral changes that were caused by co-treatment with the opioid receptor antagonist NLX in the CCP tests (Figure 1D). The PI for 30 mM morphine was significantly higher compared with the control (*p* < 0.0001). Co-treatment with 30 mM NLX significantly suppressed effects of morphine to control levels (*p* < 0.0001). No significant difference was found between the 30 mM NLX alone group and the control group. These results indicate that worms may concentration-dependently prefer morphine-conditioned cues via a specific molecule that is a common target for both morphine and NLX.

### *Npr-17* Mutant (NPR-17-Mutant) Worms do Not Prefer Morphine-Conditioned Cues

**FIGURE 2:**
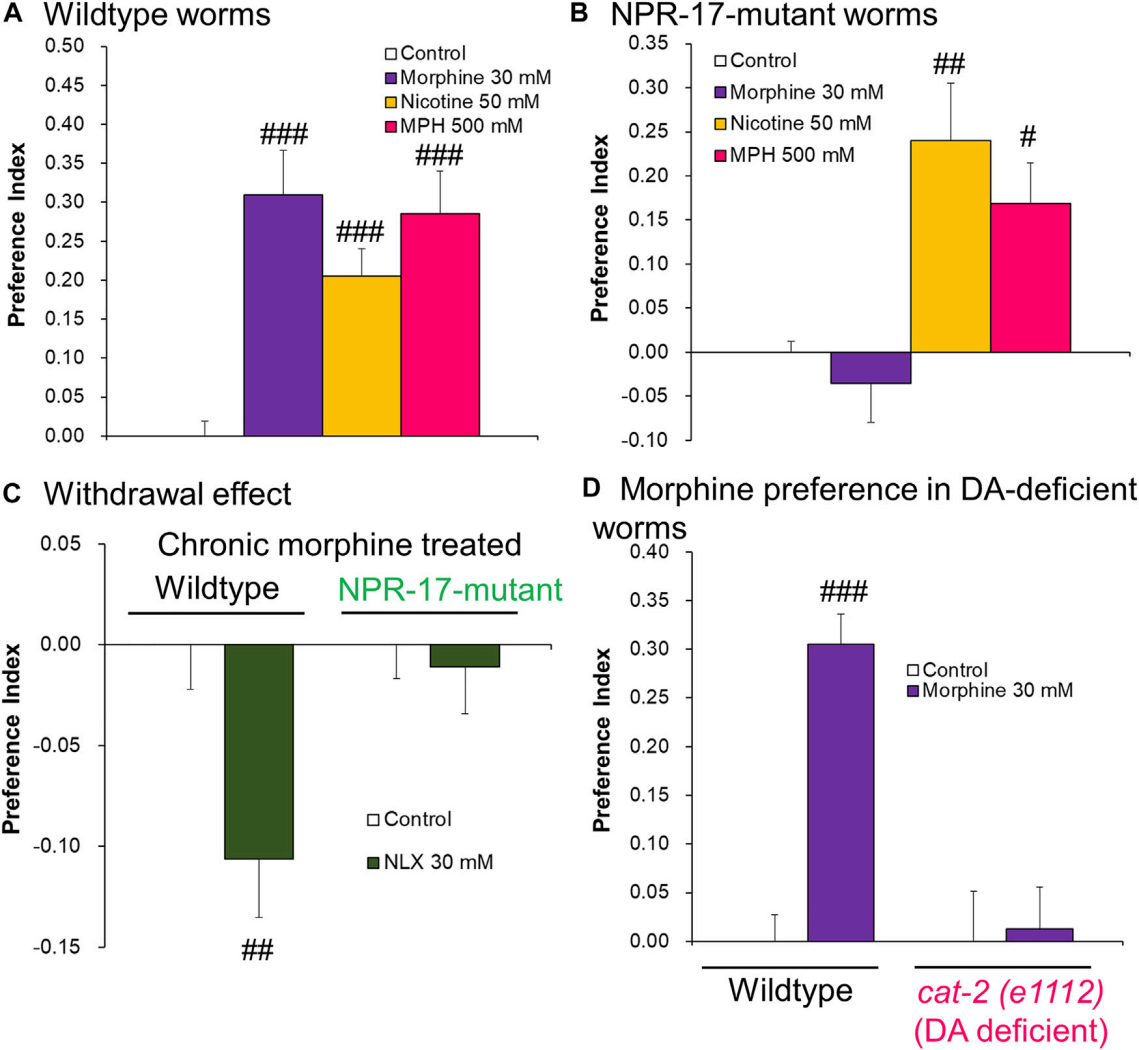
NPR-17-mutant caused the loss of morphine-induced addiction-related changes in *C. elegans.* **(A)** Preference Index in wildtype worms after 1 h of conditioning to addictive drugs in the CCP tests. One-way ANOVA: F_3,20_ = 18.42, *p* < 0.0001. (control, *n* = 14; morphine, *n* = 6; nicotine, *n* = 8; MPH, *n* = 6) **(B)** Preference Index in NPR-17-mutant worms after 1 h of conditioning to addictive drugs in the CCP tests. One-way ANOVA: F_3,28_ = 9.536, *p* = 0.0002. (control, *n* = 10; morphine, *n* = 10; nicotine, *n* = 6; MPH, *n* = 6) **(C)** Preference Index in chronic morphine-treated worms after 1 h of conditioning to 30 mM NLX in the CCP tests. Two-way ANOVA: NLX, F_1,36_ = 6.291, *p* = 0.0168; genetics, F_1,36_ = 4.181, *p* = 0.0482; interaction, F_1,36_ = 4.182, *p* = 0.0482. (*n* = 10 each) **(D)** Preference Index in wildtype and DA-deficient worms after 1 h of conditioning to 30 mM morphine in the CCP tests. Two-way ANOVA: morphine, F_1,22_ = 13.95, *p* = 0.0011; genetics, F_1,22_ = 11.85, *p* = 0.0023; interaction, F_1,22_ = 11.85, *p* = 0.0023. (control wildtype, *n* = 6; morphine wildtype, *n* = 6, control *cat-2 (e1112)*, *n* = 6; morphine *cat-2 (e1112)*, *n* = 8) ^#^ *p* < 0.05, ^##^ *p* < 0.01, ^###^ *p* < 0.001, compared with each control worm.

Morphine and NLX were previously shown to control pumping in *C. elegans* via NPR-17, an opioid-like receptor (Cheong et al., 2015), but the involvement of NPR-17 in preference-related behaviors in *C. elegans* has not been clarified. We then investigated the specific role of NPR-17 in morphine-induced preference using CCP tests. To show that the NPR-17 mutant (*tm3210*, which was reported to have a loss of function of *npr-17*; Cheong et al., 2015) does not inhibit reward function or memory/learning behavior itself but rather inhibits only the rewarding effects of morphine, we also tested nicotine and methylphenidate (MPH), two addictive substances that act via different mechanisms. Wildtype worms exhibited significantly higher approach in response to cues that were conditioned to both morphine and other addictive drugs (Figure 2A). The PIs for 30 mM morphine, 50 mM nicotine, and 500 mM MPH were significantly higher than the control in wildtype worms (morphine, *p* < 0.0001; nicotine, *p* = 0.0003; MPH, *p* < 0.0001). On the other hand, the PI for 30 mM morphine was not significantly different from the control in NPR-17-mutant worms (*p* = 0.8404, Figure 2B). Unlike morphine, the PIs for 50 mM nicotine and 500 mM MPH were significantly higher than control in NPR-17-mutant worms (nicotine, *p* = 0.0013; MPH, *p* = 0.0244). These results indicate that morphine induces preference for conditioned cues in worms via an action on NPR-17, but other addictive drugs do not.

### Worms Exhibit Aversion to NLX-Precipitated Withdrawal From Chronic Morphine Exposure

Repeated dosages of some addictive drugs, such as opioids, can lead to tolerance and physical dependence. The sudden cessation or reduction of intake can cause withdrawal symptoms. Rodents are well known to exhibit aversion to NLX (an opioid antagonist)-precipitated withdrawal from morphine treatment. Unknown is whether withdrawal symptoms are expressed in *C. elegans*. Thus, we investigated NLX-precipitated withdrawal in both wildtype and NPR-17-mutant worms after chronic 45 µM morphine (final concentration) exposure. Wildtype worms exhibited a significant decrease in the PI for the NLX-conditioned cue compared with the chronic morphine-exposed control (*p* = 0.0054), whereas NPR-17-mutant worms did not (Figure 2C). These results indicate that worms exhibit aversion to NLX-precipitated withdrawal from chronic morphine exposure via NPR-17.

### Dopamine-Deficient Worms (*Cat-2 (e1112)*) do Not Prefer Morphine-Conditioned Cues

Dopaminergic neurotransmission plays a key role in the reward system in vertebrates and *C. elegans*. Thus, we further performed CCP tests to determine the involvement of dopamine in the morphine preference in *C. elegans*. Dopamine-deficient *cat-2 (e1112)* worms (loss of function), which have a point mutation in the *cat-2* gene that encodes tyrosine hydroxylase required for dopamine synthesis, did not exhibit a preference for 30 mM morphine-conditioned cues in the CCP test (Figure 2D). These results could suggest that dopamine is necessary for morphine preference-related behavior in *C. elegans*.

## Discussion

Several researchers reported that the opioid system might exist not only in vertebrates but also in invertebrates. Endogenous opioid peptides and opioid receptors have been detected in invertebrates, and such drugs as naloxone that act on opioid receptors induce biological responses (Harrison et al., 1994). *C. elegans* was shown to have a functional opioid-like system, in which *nlp-24* encodes endogenous opioids and *npr-17* encodes an opioid receptor (Cheong et al., 2015). These authors also showed that opioid receptor agonists (i.e., the μ-opioid receptor agonist loperamide, δ-opioid receptor agonist SB205607, and κ-opioid receptor agonist U69593) activated NPR-17, and naloxone antagonized it using a heterologous expression assay in HEK293 cells (Cheong et al., 2015). Although morphine was shown to stimulate feeding in *C. elegans* and suppress overall withdrawal from noxious stimuli via an NPR-17-related pathway (Cheong et al., 2015; Mills et al., 2016), unknown was whether opioids exert addiction-like effects in *C. elegans*. In the present study, we found that morphine induced both preference and withdrawal via this opioid-like system in *C. elegans*. We also found that NPR-17 mutant worms did not exhibit preference for morphine but still exhibited preference for nicotine and MPH. Other researchers reported that NPR-17 mutant worms did not exhibit preference for alcohol (Katner et al., 2019). The findings in *C. elegans* studies are consistent with previous reports with rodents. μ-Opioid receptor knockout mice were reported to exhibit no preference for opioids or alcohol (Matthes et al., 1996; Roberts et al., 2000). μ-Opioid receptor knockout mice were also reported to exhibit preference for stimulants and nicotine, although such preference was diminished compared with wildtype mice (Berrendero et al., 2002; Hall et al., 2004). Overall, these findings suggest that the opioid system plays differential roles in behavior that is associated with preference for addictive drugs, which may be conserved across several different species, including worms, rodents, and humans.

In the present study, we used a CCP assay to evaluate preference for morphine and other addictive drugs instead of a simple chemotaxis assay, which is a type of voluntary self-exposure paradigm. Consistent with our present results, previous studies reported that *C. elegans* exhibited conditioned attraction to cues that were previously paired with cocaine and methamphetamine in a procedure that was similar to Pavlovian reward models in rodents (Katner et al., 2016; Musselman et al., 2012). Although the concentrations of drug treatments in these previous studies and the present study were relatively high because of the waxy cuticle that encases the worm and functions as a barrier to drug entry (Cheong et al., 2015; Engleman et al., 2018), the rewarding effects that were found in these studies are unlikely to be simply locomotor anesthetic or paralytic effects because *C. elegans* was not directly exposed to the test drugs during the behavioral test phase. Conditioned attraction to the cue was shown to be effective not only for stimulants, alcohol, and nicotine but also for morphine. Therefore, this method may be effective for assessing addictive drugs in general. On the other hand, this method may have problems with regard to worm starvation during the acclimation and conditioning periods. The starvation state may not always be constant and depend on the timing of the experiment and other factors. To mitigate the effects of aversion to starvation, we evaluated the approach response to conditioned cues by calculating not only the CI but also the PI. By subtracting the average CI of the control group, which was always done together in every experiment, from the CI of each experimental group, the effect of differences in starvation from experiment to experiment could be minimized. In the present study, no difference was found in the results between the CI and PI in most of the experiments. Nevertheless, there was a significant difference in the chronic morphine treatment experiment (Figure 2C) when the PI but not the CI was analyzed. Such evaluations based on the PI may allow for higher detection sensitivity by minimizing the effect of starvation in each experiment. The dopamine system is well known to play critical roles in the reward system and addiction in humans and rodents (Speranza et al., 2021). The present results could show the possibility that opioids induce preference via dopamine neurotransmission not only in vertebrates but also in *C. elegans*. Dopamine is synthesized in eight neurons in hermaphrodites: 4CEPs, 2ADEs, and 2PDEs (Chase and Koelle, 2007). NPR-17 was previously reported to be expressed in a subset of head and tail neurons, including AVG, ASIs, PVPs, PVQs, and PQR (Harris et al., 2010; Mills et al., 2016). Although these neurons do not project directly to dopamine neurons, they may control dopamine neurons via interneurons that project from neurons expressing NPR-17. NPR-17 on ASI neurons has been suggested to be important for the expression of morphine’s effects (Cheong et al., 2015; Mills et al., 2016). Whether these neurons are also important in addiction-related behaviors needs to be examined in future studies.

One of the main limitations of the present study is the absence of verification of the effects of genetic mutations. The involvement and mechanisms of NPR-17 and dopamine in morphine-induced addiction-like behaviors in *C. elegans* will be confirmed by independent verification with an alternative allele or transgenic rescue and reversal of the phenotype in future studies. Another limitation was the lack concentration-dependent effects in naloxone-precipitated withdrawal following chronic morphine exposure. In the present study, we did not determine the precise concentration or precise morphine treatment duration that cause physical dependence or the precise naloxone concentration that induces withdrawal symptoms.

In conclusion, we demonstrated that morphine caused addiction-like behaviors in *C. elegans*. These results suggest that morphine may act selectively on NPR-17 and cause preference in *C. elegans*. Consistent responses to addictive drugs across phyla lead us to hypothesize that *C. elegans* may be a useful model system to study addiction processes and screen potential treatments for substance use disorders.

## Data Availability

The original contributions presented in the study are included in the article/Supplementary Materials, further inquiries can be directed to the corresponding authors.
